# Is ERAS in laparoscopic surgery for colorectal cancer changing risk factors for delayed recovery?

**DOI:** 10.1007/s12032-016-0738-8

**Published:** 2016-02-12

**Authors:** Michał Pędziwiatr, Magdalena Pisarska, Michał Kisielewski, Maciej Matłok, Piotr Major, Mateusz Wierdak, Andrzej Budzyński, Olle Ljungqvist

**Affiliations:** 2nd Department of General Surgery, Jagiellonian University Medical College, Kraków, Poland; Department of Endoscopic, Metabolic and Soft Tissue Tumors Surgery, Kopernika 21, 31-501 Kraków, Poland; Department of Surgery, Faculty of Medicine and Health, School of Health and Medical Sciences, Örebro University, Örebro, Sweden

**Keywords:** Enhanced recovery after surgery, Fast-track surgery, Laparoscopy, Perioperative care, Compliance with protocol, Colorectal cancer

## Abstract

There is evidence that implementation of enhanced recovery after surgery (ERAS) protocols into colorectal surgery reduces complication rate and improves postoperative recovery. However, most published papers on ERAS outcomes and length of stay in hospital (LOS) include patients undergoing open resections. The aim of this pilot study was to determine the factors affecting recovery and LOS in patients after laparoscopic colorectal surgery for cancer combined with ERAS protocol. One hundred and forty-three consecutive patients undergoing elective laparoscopic resection were prospectively evaluated. They were divided into two subgroups depending on their reaching the targeted length of stay—LOS (75 patients in group 1—≤4 days, 68 patients in group 2—>4 days). A univariate and multivariate logistic regression analysis was performed to assess for factors (demographics, perioperative parameters, complications and compliance with the ERAS protocol) independently associated with LOS of 4 days or longer. The median LOS in the entire group was 4 days. The postoperative complication rate was higher (18.7 vs. 36.7 %), and the compliance with ERAS protocol was lower (91.2 vs. 76.7 %) in group 2. There was an association between the pre- and postoperative compliance and the subsequent complications. In uni- and multivariate analysis, the lack of balanced fluid therapy (OR 3.87), lack of early mobilization (OR 20.74), prolonged urinary catheterization (OR 4.58) and use of drainage (OR 2.86) were significantly associated with prolonged LOS. Neither traditional patient risk factors nor the stage of the cancer was predictive of the duration of hospital stay. Instead, compliance with the ERAS protocol seems to influence recovery and LOS when applied to laparoscopic colorectal cancer surgery.

## Introduction

Perioperative care programs in elective colorectal surgery based on Enhanced Recovery After Surgery^®^ (ERAS) Society recommendations have been shown to reduce the length of hospital stay (LOS) and lower the complication rate in colorectal surgery [1–4]. Some elements of the protocol, such as balanced fluid therapy, multimodal analgesia, metabolic management including early oral feeding and early mobilization, have been reported as the ones with specific importance [4, 5]. When these elements are implemented, the reduction in the recovery time to a median LOS as short as 2–3 days for open colonic resections was noted [6, 7]. However, the commonly reported stays within an ERAS protocol are reduced from 9–10 to 5–6 days [3, 8–10]. In addition, the use of the laparoscopic technique in ERAS has been shown to lead to even faster recovery, decreased complication rate and shorter LOS in most studies [8, 9, 11], but not in all cases [12]. Better optimization of the ERAS pathway together with adequate organizational arrangements at the hospital, combined with laparoscopic surgery, allows a further reduction in stay down to 1 day in a selected group of patients [13].

However, the goal of modern perioperative care is not primarily to minimize the LOS but rather to improve the quality of the recovery [14]. The improved and faster recovery will also allow for earlier discharges from hospitals. However, very short postoperative hospital stay may result in more readmissions. In the early work of Kehlet readmission after ultra-short stays was as high as 20 % [15]. Later research showed that improved adherence to ERAS protocols resulted in fewer readmissions despite shorter stay after open elective colorectal surgery [3]. Nevertheless, later data also showed that using ERAS fully or just using more ERAS elements as assembled in several meta-analyses since 2010 results in fewer complications and shorter stays in hospital [3, 4, 11, 16, 17].

Minimally invasive techniques offer less surgically induced trauma and influence other postoperative care elements [18]. Most of studies focus on short-term outcomes such as LOS, complications and readmissions [8, 19]. So far, little is known whether the combination of ERAS and minimally invasive surgery impacts also the influence of traditional risk factors on short-term surgical outcomes. According to previously published analyses, such combination may indeed diminish the negative effect of some demographic parameters (age, comorbidities, ASA grade) in postoperative period [20]. However, this topic is still less studied and therefore requires further investigation.

## Aim

The aim of the current pilot study was to analyze factors affecting recovery and length of stay in patients after laparoscopic colorectal surgery for cancer treated according to the protocol based on the ERAS Society guidelines [1, 2].

## Materials and methods

The analysis included prospectively collected data from consecutive patients electively operated for colorectal cancer in the years 2013–2014. During this time, the standard primary surgical approach at the unit was that all elective patients were operated using laparoscopic surgery, and the perioperative care was based on preestablished ERAS protocol consisting of 16 items (Table 1). Its principles and criteria for discharge from the hospital were based on the ERAS Society guidelines [21, 22].

**Table 1:** ERAS protocol used in our department

1. Preoperative counseling and patient’s education
2. No bowel preparation (oral lavage in the case of low rectal resection with TME and defunctioning loop ileostomy)
3. Preoperative carbohydrate loading (400 ml of Nutricia preOp^®^ 2 h prior surgery)
4. Antithrombotic prophylaxis (Clexane^®^ 40 mg sc. starting in the evening prior surgery)
5. Antibiotic prophylaxis (preoperative cefuroxime 1.5 g + metronidazole 0.5 g iv. 30–60 min prior surgery)
6. Laparoscopic surgery
7. Balanced intravenous fluid therapy (<2500 ml intravenous fluids during the day of surgery, <150 mmol sodium)
8. No nasogastric tubes postoperatively
9. No drains left routinely for colonic resections, one drain placed for <24 h in case of TME
10. TAP block and standard anesthesia protocol
11. Avoiding opioids, multimodal analgesia (oral when possible—paracetamol 4 × 1 g, ibuprofen 2 × 200 mg, metamizole 2 × 500 mg or ketoprofen 2 × 100 mg)
12. Prevention of postoperative nausea and vomiting (PONV) (dexamethasone 8 mg iv., ondansetron 8 mg iv., metoclopramide 10 mg iv.)
13. Postoperative oxygenation therapy (4–6 l/min)
14. Early oral feeding (oral nutritional supplement 4 h postoperatively—Nutricia Nutridrink^®^ or Nestlé Impact^®^, light hospital diet and oral nutritional supplements on the first postoperative day, full hospital diet in the second postoperative day)
15. Urinary catheter removal on the first postoperative day
16. Full mobilization on the first postoperative day (getting out of bed, going to toilette, walking along the corridor, at least 4 h out of bed)

Patients submitted initially for open or emergency surgery or those with complex cancer who required multiorgan resection, and patients treated with endoscopic techniques using the hybrid TaTME technique were excluded from the studied group.

**Fig. 1:**
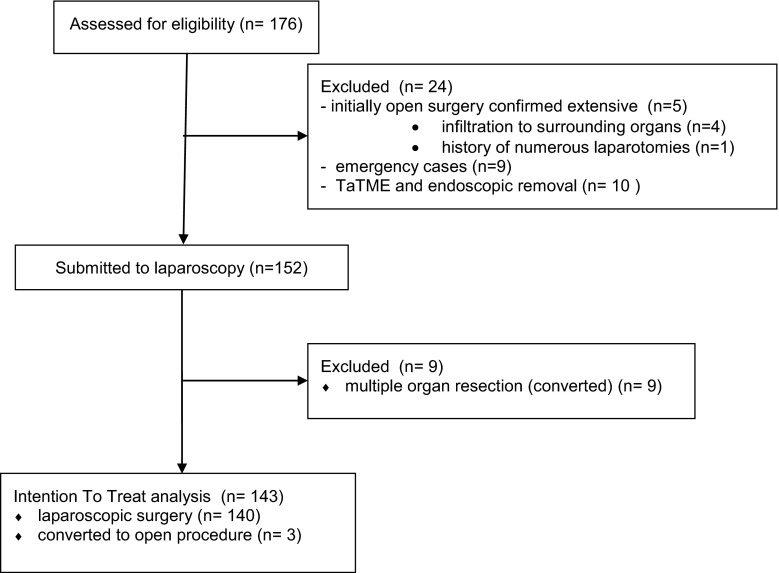
Patient ITT flowchart

During the time period of the study, a total of 176 patients with colorectal cancer were operated in the department. Twenty-four patients did not meet inclusion criteria. Out of the remaining 152 patients, 12 were converted. The reasons for conversion were tumor infiltration to surrounding organs (nine cases that were excluded from the analysis) or other technical difficulties (three cases that were included in the intention to treat analysis). Overall 143 patients (68 women and 75 men) underwent laparoscopic resections (Fig. 1).

Their mean age was 66.8 years (27–94 years). One hundred patients underwent colonic resection, and 43 had rectal resection. The demographic analysis of the group is shown in Table 2.

**Table 2:** Demographic analysis of patient groups

Parameter	All patients	Group 1 (≤4 days)	Group 2 (>4 days)	*p* value
Number of patients, *n* (%)	143	75 (52.4 %)	68 (47.6 %)	–
Females, *n* (%)	68 (47.6 %)	39 (52 %)	29 (42.6 %)	0.26
Males, *n* (%)	75 (52.4 %)	36 (48 %)	39 (57.4 %)	
Mean age, years ± SD	66.8 ± 12.6	65.9 ± 12.9	67.3 ± 12.3	0.35
BMI, kg/m^2^ ± SD	25.9 ± 4.9	26.5 ± 5.3	25.2 ± 4.5	0.24
ASA 1, *n* (%)	5 (3.5 %)	4 (5.3 %)	1 (1.5 %)	0.14
ASA 2, *n* (%)	87 (60.9 %)	44 (58.7 %)	43 (63.2 %)	
ASA 3, *n* (%)	47 (32.8 %)	23 (30.7 %)	24 (35.3 %)	
ASA 4, *n* (%)	4 (2.8 %)	4 (5.3 %)	0 (0 %)	
Any comorbidity, *n* (%)	109 (76.2 %)	54 (72.0 %)	55 (80.9 %)	0.21
Cardiovascular, *n* (%)	52 (36.4 %)	25 (33.3 %)	27 (39.7 %)	0.92
Hypertension, *n* (%)	74 (51.7 %)	39 (52.0 %)	35 (51.5 %)	0.95
Diabetes, *n* (%)	28 (19.6 %)	15 (20 %)	13 (19.1 %)	0.89
Pulmonary, *n* (%)	21 (14.7 %)	9 (12.0 %)	12 (17.6 %)	0.34
Renal, *n* (%)	12 (8.4 %)	7 (9.3 %)	5 (7.4 %)	0.67
Liver, *n* (%)	5 (3.5 %)	1 (1.3 %)	4 (5.9 %)	0.14
Distance from the place of residence <50 km, *n* (%)	103 (72 %)	52 (69.3 %)	51 (75.0 %)	0.33
Distance from the place of residence >50 km, *n* (%)	40 (28 %)	23 (30.1 %)	17 (25.0 %)	
Colonic resection, *n* (%)	100 (69.9 %)	56 (74.7 %)	44 (64.7 %)	0.19
TME, *n* (%)	43 (30.1 %)	19 (25.3 %)	24 (35.3 %)	0.50
AJCC Stage I, *n* (%)	45 (31.5 %)	27 (36 %)	18 (26.5 %)	
AJCC Stage II, *n* (%)	49 (34.2 %)	26 (34.7 %)	23 (33.8 %)	
AJCC Stage III, *n* (%)	34 (23.8 %)	16 (21.3 %)	18 (26.5 %)	
AJCC Stage IV, *n* (%)	15 (10.5 %)	6 (8 %)	9 (13.2 %)	
Mean length of hospital stay, days (range)	5.5 (2–40)	2.9 (2–4)	8.4 (5–40)	–
Median length of hospital stay, days (IQR)	4 (3–7)	3 (2–3)	7 (5–8)	–
Mean operative time, min ± SD	185.1 ± 62.2	185.4 ± 51.3	184.8 ± 73.8	0.49
Mean intraoperative blood loss, ml ± SD	90.2 ± 79.5	84.3 ± 74.1	97 ± 86.1	0.36
Conversion, *n* (%)	3 (2.1 %)	0	3 (4.4 %)	–

*AJCC* American Joint Committee on Cancer, *ASA* American Society of Anaesthesiologists, *SD* standard deviation, *IQR* interquartile range

We analyzed the influence of the following factors on LOS (primary length of stay, excluding readmissions): gender; age; BMI; ASA (American Society of Anaesthesiologists), physical status; the presence of preoperative comorbidities; type of surgery (colonic resection vs. rectal resection with total mesorectal excision, TME); stage of cancer; distance between the hospital and place of residence; operative time; and intraoperative blood loss. Moreover, the compliance with ERAS protocol was also analyzed, taking into consideration its selected items: mechanical bowel preparation, preoperative carbohydrate loading (CHO loading), balanced fluid therapy (<2500 ml intravenous fluids), early mobilization on the day of surgery (all patients are actively encouraged by nursing staff to be mobile), early introduction of oral feeding—each patient received an oral nutritional supplement in the afternoon on the day of the surgery and light hospital diet on the first postoperative day followed by a full diet on the second postoperative day, use of drains, prolonged (>24 h) urinary catheterization and use of opioids (administered only if the standard regimen was not sufficient). Compliance was calculated as the number of pre- and intraoperative interventions fulfilled/13 (number of protocol elements included) similarly to Gustafsson [3]. Additionally, we analyzed the rate of postoperative complications and the readmission rate.

A standardized anesthetic protocol was used in all patients: Antimicrobial prophylaxis (cefuroxime 1.5 g iv. and metronidazole 0.5 g iv.) was administered 30–60 min prior the first incision. Following preoxygenation, patients were given fentanyl, 200–500 mcg iv. General anesthesia was induced with propofol, 100–300 mg iv. Tracheal intubation was facilitated with rocuronium. General anesthesia was maintained with sevoflurane and fentanyl (100 mcg bolus every 30 min). Rocuronium was used for muscle relaxation. Perioperatively, patients received postoperative nausea and vomiting prophylaxis (dexamethasone, ondansetron). Colonic or rectal resections were performed laparoscopically according to all oncological principles as described elsewhere [23]. Complications were graded using the Clavien–Dindo classification [24]. Readmission was defined as any patient hospitalization related to the surgery within 30 days after being discharged home.

The study obtained the ethical approval from the local ethics review committee and has been performed in accordance with the ethical standards laid down in the 1964 Declaration of Helsinki and its later amendments. It was registered under NCT02527967 (ClinicalTrials.gov). Informed consent was obtained from all patients before surgery.

StatSoft Statistica v.10 was used for statistical analysis. For the purposes of further analyses, the entire group of patients was divided into two subgroups depending on the length of their hospital stay. On admission, every patient received the information about the target length of stay of 4 days. Group 1 consisted of patients whose hospital stay was shorter or equal to the target LOS (≤4 days). In group 2 were patients whose hospital stay was longer than 4 days. Additionally, the entire group of patients was divided according to occurrence of complications. The study of categorical variables used the Chi-square test of independence. In the case of non-normally distributed quantitative variables, Mann–Whitney U test was used. An univariate logistic regression analysis of individual demographic and perioperative parameters was undertaken to assess factors influencing prolonged LOS as well as occurrence of complications. Finally, the variables in the univariate logistic regression analysis that had a significant impact on the length of hospital stay were used to build a multivariate logistic regression model. Results were considered statistically significant when *p* value was found to be <0.05.

## Results

The median length of hospital stay in the entire group was 4 days, and this was the planned LOS before the study was initiated. Therefore, two subgroups were created (cutoff LOS 4 days) for the purpose of further statistical analyses. The analysis of demographic parameters (including the location and stage of cancer as well as type of surgery and comorbidities) showed no significant differences between groups as presented in Table 2. In contrast, we have found some differences in the use of pre- and intraoperative ERAS protocol items (Table 3). Moreover, there was also a significant difference in overall compliance with the protocol (91.2 ± 9.6 vs. 76.7 ± 13.6, *p* = 0.00001).

**Table 3:** Perioperative parameters in analyzed groups

Parameter	Group 1 (≤4 days)	Group 2 (>4 days)	*p* value
Mechanical bowel preparation, *n* (%)	25 (33.3 %)	36 (52.9 %)	0.02
Preoperative CHO loading, *n* (%)	58 (77.3 %)	37 (54.4 %)	0.006
Balanced fluid therapy, *n* (%)	69 (92 %)	42 (61.8 %)	0.00002
Peritoneal drainage, *n* (%)	14 (18.7 %)	38 (55.9 %)	0.00001
Prolonged (>24 h) catheterization after surgery, *n* (%)	5 (6.7 %)	19 (27.9 %)	0.0003
Stoma formation, *n* (%)	8 (10,6 %)	14 (20.6 %)	0.10
Postoperative use of opioids, *n* (%)	26 (34.7 %)	27 (39.7 %)	0.40
Tolerance of full oral diet in the first postoperative day, *n* (%)	58 (77.3 %)	37 (54.4 %)	0.005
Mobilization on the day of the surgery, *n* (%)	74 (98.6 %)	42 (61.8 %)	0.00001

### Complications

The overall complication rate was 27.3 % (18.7 vs. 36.8 % in group 1 and 2, *p* = 0.02). They are summarized in Table 4. We observed a significant difference in compliance between patients with and without complications when calculated for the entire group (86.5 vs. 79.1 %, *p* = 0.005). Readmission occurred in eight (5.6 %) patients (six patients in group 1 and two patients in group 2, *p* = 0.19). Using univariate regression analysis, we observed that only bowel preparation (OR 3.15, 95 % CI 1.45–6.86, *p* = 0.004) and non-balanced fluid therapy (OR 2.99, 95 % CI 1.28–6.96, *p* = 0.012) were independent predictors of complications in our group. The remaining parameters such as: CHO loading (OR 1.49, 95 % CI 0.68–3.27, *p* = 0.31), tolerance of full oral diet in the first postoperative day (OR 1.71, 95 % CI 0.79–3.72, *p* = 0.17), mobilization on the day of surgery (OR 1.29, 95 % CI 0.50–3.29, *p* = 0.6), peritoneal drainage (OR 1.35, 95 % CI 0.63–2.92, *p* = 0.44), prolonged urinary catheterization (OR 1.43, 95 % CI 0.55–3.73, *p* = 0.46), type of surgery (OR 1.79, 95 % CI 0.82–3.94, *p* = 0.14), use of opioids (OR 0.94, 95 % CI 0.43–2.08, *p* = 0.88) as well as sex (OR 1.01, 95 % CI 0.47–2.18, *p* = 0.98), age (OR 0.86, 95 % CI 0.41–1.84, *p* = 0.7), comorbidities (OR 1.23, 95 % CI 0.5–3.05, *p* = 0.65) and stage of disease (OR 0.72, 95 % CI 0.19–2.78, *p* = 0.62) had no influence on complications. Since only two parameters were statistically significant in univariate logistic regression analysis, we decided not to follow with the multivariate logistic regression model.

**Table 4:** Types of complications in both groups

Clavien–Dindo classification	Complications	Group 1 (≤4 days)		Group 2 (>4 days)		*p* value
III B	Perforation of transverse colon from Veress needle (relaparoscopy, suturing)	0/14 (0 %)	3 (4.0 %)	1/25 (4 %)	6 (8.8 %)	0.51
	Perforation of small intestine (relaparotomy, resection)	0/14 (0 %)		1/25 (4 %)		
	Peristomal fistula (correction under general anesthesia)	0/14 (0 %)		1/25 (4 %)		
	Trocar-related abdominal wall bleeding (relaparoscopy)	1/14 (7 %)		0/25 (0 %)		
III A	Bleeding from anastomosis suture line (controlled endoscopically)	2/14 (14 %)		0/25 (0 %)		
	Anastomosis leakage (treated with Endo-SPONGE^®^)	0/14 (0 %)		3/25 (12 %)		
II	Intraperitoneal hematoma	0/14 (0 %)	1 (1.3 %)	1/25 (4 %)	5 (7.3 %)	
	Infectious diarrhea (C. difficile)	0/14 (0 %)		1/25 (4 %)		
	Pneumonia	0/14 (0 %)		1/25 (4 %)		
	Urinary tract infection	1/14 (7 %)		2/25 (8 %)		
I	Surgical site infection	3/14 (22 %)	10 (13.3 %)	5/25 (20 %)	14 (20.6 %)	
	Non-infectious diarrhea	1/14 (7 %)		0/25 (0 %)		
	Postoperative nausea and vomiting	5/14 (36 %)		7/25 (28 %)		
	Arrhythmia	1/14 (7 %)		1/25 (4 %)		
	Postoperative confusion	0/14 (0 %)		1/25 (4 %)		

### Comorbidities

One hundred and nine (76.2 %) patients had at least one comorbidity. There were no differences in the types of comorbidity between groups 1 and 2. We did not observe any influence of the presence of one or more comorbidities on LOS or complications except for the presence of cardiovascular disease that was significantly more prevalent in the patients without complications (Table 5).

**Table 5:** Comorbidities and their relation to length of stay and complications

Length of stay					
	All patients	Group 1 (≤4 days)	Group 2 (>4 days)	*p* value	
Comorbidity					
Any comorbidity	109 (76.2 %)	54 (72.0 %)	55 (80.9 %)	0.21	
Cardiovascular	52 (36.4 %)	25 (33.3 %)	27 (39.7 %)	0.92	
Hypertension	74 (51.7 %)	39 (52.0 %)	35 (51.5 %)	0.95	
Diabetes	28 (19.6 %)	15 (20 %)	13 (19.1 %)	0.89	
Pulmonary	21 (14.7 %)	9 (12.0 %)	12 (17.6 %)	0.34	
Renal	12 (8.4 %)	7 (9.3 %)	5 (7.4 %)	0.67	
Liver	5 (3.5 %)	1 (1.3 %)	4 (5.9 %)	0.14	
Number of comorbidities					
0	34 (23.8 %)	21 (28.0 %)	13 (19.1 %)	0.58	
1	58 (40.5 %)	27 (36.0 %)	31 (45.6 %)		
2	24 (16.8 %)	13 (17.4 %)	11 (16.2 %)		
3	23 (16.1 %)	12 (16.0 %)	11 (16.2 %)		
4	3 (2.1 %)	1 (1.3 %)	2 (2.9 %)		
5	1 (0.7 %)	1 (1.3 %)	0		

Complications				
	All patients	Without complications	With complications	*p* value
Parameter				
Any comorbidity	109 (76.2 %)	79 (76.0 %)	30 (76.9 %)	0.90
Cardiovascular	52 (36.4 %)	43 (41.3 %)	9 (23.1 %)	0.04
Hypertension	74 (51.7 %)	53 (51.0 %)	21 (53.8 %)	0.76
Diabetes	28 (19.6 %)	20 (19.2 %)	8 (20.5 %)	0.86
Pulmonary	21 (14.7 %)	18 (17.3 %)	3 (7.7 %)	0.13
Renal	12 (8.4 %)	11 (10.6 %)	1 (2.6 %)	0.09
Liver	5 (3.5 %)	4 (3.9 %)	1 (2.6 %)	0.70
Number of comorbidities				
0	34 (23.8 %)	25 (24.0 %)	9 (23.1 %)	0.26
1	58 (40.5 %)	37 (35.6 %)	21 (53.8 %)	
2	24 (16.8 %)	19 (18.3 %)	5 (12.8 %)	
3	23 (16.1 %)	19 (18.3 %)	4 (10.3 %)	
4	3 (2.1 %)	3 (2.9 %)	0	
5	1 (0.7 %)	1 (0.9 %)	0	

### Length of stay

In the current patient group, age, gender, ASA, BMI, presence of comorbidities, distance from the place of residence, cancer stage, presence of a stoma or use of opioids had no effect on primary length of hospital stay. In the univariate logistic regression analysis, it was found, however, that presence of postoperative complications, bowel preparation, lack of CHO loading, non-balanced fluid therapy, poor tolerance of early oral diet, lack of mobilization on the day of surgery, peritoneal drainage, >24-h urinary catheterization, were all related to prolonged LOS (Table 6).

**Table 6:** Uni- and multivariate logistic regression analysis (adjusted for type of surgery colon/rectum) of the parameters affecting prolonged hospitalization (*R* ^2^ Nagelkerke = 0.46)

Parameter	Univariate logistic regression			Multivariate logistic regression		
	OR	95 % CI	*p* value	OR	95 % CI	*p* value
Mechanical bowel preparation	2.25	1.14–4.45	0.02	1.14	0.42–3.11	0.79
No preoperative CHO loading	2.75	1.32–5.73	0.007	1.23	0.41–3.66	0.70
Non-balanced fluid therapy	6.85	2.57–18.21	0.00015	3.87	1.02–14.71	0.046
Peritoneal drainage	5.62	2.62–12.04	0.00001	2.86	1.01–8.14	0.048
Prolonged (> 24 h) catheterization	5.57	1.92–16.17	0.002	4.58	1.33–15.74	0.02
Tolerating oral diet on the first postoperative day	2.77	1.33–5.74	0.007	1.01	0.33–3.10	0.99
Mobilization on the day of surgery	45.81	5.89–356.12	0.0003	20.74	2.25–191.30	0.008
Postoperative complications	2.38	1.10–5.14	0.03	2.00	0.72–5.59	0.18
Colon/rectum	1.61	0.78–4.32	0.19	1.15	0.37–3.56	0.81

In turn, the multivariate logistic regression model (*R*^2^ Nagelkerke coefficient of determination = 0.46) only fluid overload, lack of mobilization on the day of surgery, prolonged (>24 h) urinary catheterization and peritoneal drainage remained significant factors prolonging LOS (Table 6).

## Discussion

In this pilot study of patients undergoing laparoscopic colorectal resections combined with the ERAS protocol, we found that many of the traditional demographic factors such as age, comorbidity, ASA stage seem to have little or no impact on short-term outcomes. When using the multivariate regression model adjusted for the type of surgery, we observed that it was a low compliance with perioperative ERAS care elements that prolonged LOS rather than factors traditionally reported to prolong recovery. The study showed that if the patients were not mobilized on the day of surgery, received non-balanced fluid therapy, had prolonged catheterization or peritoneal drainage, they also stayed longer in the hospital. When comparing the patients leaving within target LOS of 4 days with those staying longer, it was found that overall differences in compliance with the ERAS protocol and several of the single elements as well as complication rates differed between these two patient groups. Similar to several previous reports, we also found an association between ERAS protocol compliance and complications [3, 9].

In contrast, we failed to demonstrate any influence of demographic parameters and classical risk factors on the development of complications. This is likely to be explained by the low level of stress inflicted when the stress reducing protocol elements of the ERAS protocol and laparoscopic surgery is combined [25]. In this study, we did not investigate potential mechanisms behind this finding. However, previous studies suggest that reducing the stress response to surgery, minimizing the inflammatory responses, controlling pain relief and retaining homeostasis for metabolism and fluid balance are all likely to contribute to the finding that these classical risk factors are of less importance [26–29].

Our study group consisted of consecutive cases submitted to minimally invasive colorectal resection in an ERAS environment. Although laparoscopy is gaining momentum and the rate of these procedures increases worldwide, the data comprising only laparoscopic cases in ERAS are sparse. According to Khan et al., no further benefit is obtained by the inclusion of laparoscopic surgery in ERAS protocols. However, the evidence was limited [30]. In 2011, Vlug et al. [8] published the first randomized controlled trial, showing that in ERAS environment laparoscopic surgery can indeed bring benefits such as shorter LOS and reduced morbidity. It was later confirmed by Kennedy et al. [19] in EnROL trial. However, most of the current studies in large bowel surgery comprise heterogeneous groups of both open and laparoscopic procedures or open series only. The conversion rate of 2.1 % is relatively low comparing to other studies on laparoscopic surgery [20, 31–34]. The mean LOS (5.5 days) as well as the complication rate (27.3 %) of the entire group reported in this study is in line with previously published groups [35–37].

The mean overall compliance with the ERAS protocol of 83 % is similar if not higher than presented in other studies [3, 20, 32]. The link between the adherence to the protocol and outcomes was extensively studied by Gustafsson [3] in open colorectal resections, who showed that improving compliance may result in reducing postoperative morbidity and thus shortening of LOS. Our results in laparoscopic resections are consistent with these observations. We found that compliance in group 1 was significantly higher than in group 2 with longer stay. This supports the notion that the length of hospital stay is influenced by pre- and intraoperative ERAS parameters. These are also the treatment choices that are mostly influenced by the team treating the patient, and to a lesser extent dependent on patient factors.

Perhaps the most interesting finding in this group of patients was that none of the classical demographic parameters analyzed had any effect on prolonged hospitalization. Thus, neither age and comorbidities nor ASA grade had any impact on length of stay or complications. Similar conclusions were drawn by Smart et al. [20] in laparoscopic colorectal surgery. Our observation, however, is in contrary to those presented in other studies comprising patients undergoing open or minimally invasive colorectal resection. Hendry reported that age, male sex and rectal surgery influenced LOS [38]. Although most studies found that serious comorbidities (ASA III–IV) are indeed a risk factor for worse outcomes, the link between the presence, number as well as their types and complication rate or prolonged LOS was not confirmed in our analysis [35, 38, 39]. In large series from Denmark employing ERAS methodology in hip and knee replacement, it was showed that age is less of a factor for length of stay and even patients older than 85 years have a median stay of 4 days [40]. The same group also showed that major risk factors such as diabetes have much less impact, if any, with modern enhanced recovery care [41].

In a recent study from the ERAS Compliance Group, balanced fluid therapy was one of the strongest factors influencing postoperative outcomes [9]. The benefits of appropriate fluid management have been shown repeatedly [42–45]. In our study, not maintaining balanced fluid therapy was associated with an almost four times higher risk of prolonged stay and higher complication rates. In 62.9 % of all patients, fluids were stopped within 24 postoperatively. It was in 70.7 % patients from group 1 and in 54.4 % from group 2. According to our protocol, we do not continue infusions postoperatively when not needed. Prolonged intravenous fluid therapy was mostly due to inability to tolerate oral fluids (complications such as PONV, ileus). However, in some patients, we had to come back to iv. fluids in subsequent days due to delayed recovery of the gastrointestinal function.

Several studies have shown that keeping high compliance with the ERAS protocol results in a reduction in morbidity [3, 9, 46]. We found that certain factors were of specific importance for the time of recovery. Tolerating diet on the day of surgery was one of them. An attempt to introduce an oral diet on the day of the surgery was made in all patients, tolerating early feeding was much better in the group that managed early discharge compared to those with longer stay (77.3 vs. 55.4 %, *p* = 0.0051). Not tolerating early feeding was associated with prolonged stay. Mobilization on the day of surgery was another factor that affected recovery time. Gustafsson et al. [47] made the comparison of open and laparoscopic rectal surgery in terms of early mobilization within an ERAS protocol and showed benefits of the latter. All patients in the group with early discharge (with the exception of one patient who initially was on the wheelchair) were mobilized on the day of surgery. In contrast, in group 2 only 60 % of patients were ambulated. As mentioned above, while early oral diet tolerance and mobilization may be regarded as outcomes and result of care given earlier during the patients pathway, they can serve as early predictors of delayed recovery [20, 34, 48].

In hospitals where ERAS protocol is fully implemented, mean LOS can be shortened to 1–4 days in select groups of patients [13, 37]. In this situation, any complication will inevitably lead to longer hospital stay. Our observations are consistent with the findings of other authors in this respect [34, 35, 39, 49]. In the group keeping to short LOS complications were mostly Clavien–Dindo grade 1, and all but one case were treated conservatively. These problems had minimal impact on recovery and did not necessarily prolong hospitalization. In contrast, the prolonged length of stay in the delayed discharge group was explained to a large extent by the more severe types of complications. However, when they were included in the multivariate model, they were not significant predictors of prolonged LOS. The relatively small group of patients with complications and the fact that there were also other factors that had greater impact on outcomes may explain this divergence between uni- and multivariate logistic regression analysis. In this group, there were more complex problems and many complications deemed either another surgical/endoscopic intervention or longer conservative treatment.

The data in the literature on the location and stage of cancer influencing LOS are contradictory [20, 35, 38, 39, 50–52]. In our analysis, the percentage of patients with rectal cancer in group 2 was indeed greater, although the difference was not significant. It is reasonable to assume that the type of surgery (colonic or rectal) influenced outcomes to some extent, and for that reason, this was included in the analysis and showed no impact. Although it has been previously shown that the stage of cancer influences LOS in open surgery with traditional care, we were unable to confirm such relationship in our laparoscopic group with ERAS protocol [53]. In our model, we were able to explain almost 50 % of the variation in length of stay with the factors identified. This is a reasonable level, but there remain many factors to be taken into account to understand the entire picture. One such factor that we did not study was the capacity of the receiving end that is the care or support at home or availability of the caretakers after discharge.

## Conclusions

Our observations in patients undergoing laparoscopic colorectal surgery in an ERAS environment indicate that delayed recovery and prolonged hospital stay are associated with lack of compliance with some ERAS protocol elements such as maintaining fluid balance, mobilization postoperatively as well as remaining catheters and drains, and the development of complications. In this context, patient demographic characteristics, comorbidities, anesthetic risk or stage of the disease was not predictive of the duration of hospital stay or development of complications.
